# Posterior Migration of a Descemet Membrane Endothelial Keratoplasty Graft Associated With Macular Proliferative Changes

**DOI:** 10.7759/cureus.100897

**Published:** 2026-01-06

**Authors:** Naoki Nezu, Toshiki Shimizu, Yorihisa Kitagawa, Takahiko Hayashi, Satoru Yamagami

**Affiliations:** 1 Ophthalmology, Nihon University School of Medicine, Tokyo, JPN

**Keywords:** dmek, graft dislocation, macular proliferation, vitrectomy, vitreous cavity

## Abstract

A 79-year-old male underwent descemet membrane endothelial keratoplasty (DMEK) for bullous keratopathy. Intraoperatively, the intraocular lens (IOL) became unstable during graft manipulation, and the graft was lost from the anterior chamber. Postoperative examination failed to identify the graft. Two weeks later, optical coherence tomography (OCT) showed macular proliferative changes without hemorrhage or peripheral ischemia. Subsequent pars plana vitrectomy revealed the DMEK graft in the vitreous cavity, associated with fibrovascular proliferation. The graft was successfully removed. This case demonstrates a rare complication of DMEK and emphasizes the need for vigilance in eyes with compromised posterior capsule or zonules.

## Introduction

Descemet membrane endothelial keratoplasty (DMEK) has become the preferred surgical technique for the management of corneal endothelial dysfunction due to its rapid visual recovery and favorable graft survival compared with other forms of endothelial keratoplasty [1-3]. However, the procedure can be technically challenging, particularly in eyes with altered anterior or posterior segment anatomy. Eyes that have undergone prior vitrectomy represent a unique surgical subgroup, as the absence of vitreous support may predispose the graft to abnormal intraocular behavior, including posterior migration [4].

Although posterior dislocation of endothelial keratoplasty grafts has been reported, such events remain extremely rare, and the majority of published cases focus primarily on graft localization and retrieval rather than secondary posterior segment complications. Herein, we report a rare case of posterior migration of a DMEK graft into the vitreous cavity associated with early localized macular proliferative changes, highlighting the importance of comprehensive posterior segment evaluation and timely multidisciplinary management.

## Case presentation

A 79-year-old male with no systemic medical history was referred for progressive visual decline and corneal edema in the right eye. His ocular history included cataract surgery, anterior uveitis treated with topical steroids, and vitrectomy for premacular membrane removal.

**Figure 1:**
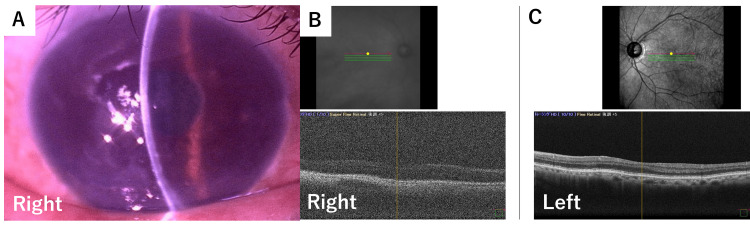
Preoperative anterior segment findings. (A) Slit-lamp photograph demonstrating diffuse corneal edema without inflammatory cells in the anterior chamber. The posterior chamber intraocular lens (PCIOL) appears stable and is fixated in the capsular bag. (B) Optical coherence tomography (OCT) demonstrated no abnormal macular findings, including cystoid macular edema or proliferative changes. (C) Optical coherence tomography (OCT) of the contralateral eye demonstrated no abnormal findings.

At presentation, best-corrected visual acuity washand movements close to the face in the right eye and 20/20 in the left eye. Slit-lamp examination showed the existence of pseudoexfoliation (PEX) syndrome, and diffuse corneal edema without inflammatory cells (Figure 1A). The posterior chamber intraocular lens (one-piece PCIOL, in-the-bag) appeared stable. Despite difficulty in precise evaluation in retina, we could see light reflex, and optical coherence tomography (OCT) showed a regular shape without cystoid macular edema (CME) or proliferative findings in the right eye (Figure 1B) and the left eye (Figure 1C). The intraocular pressure was 14 mmHg in the right eye and 17 mmHg in the left eye. Specular microscopy revealed an unmeasurable endothelial cell density. The patient was diagnosed with bullous keratopathy, and descemet membrane endothelial keratoplasty (DMEK) was scheduled.

Standard DMEK was performed. After stripping the host Descemet membrane, an 8.0-mm donor graft was injected into the anterior chamber. During graft unfolding and air tamponade, the PCIOL became unstable, showed instability (donesis) and shifted from its original position during graft manipulation. During this maneuver, the DMEK graft was lost and could no longer be visualized. Despite a thorough intraoperative search, the graft could not be identified, and the procedure was aborted.

Postoperatively, the cornea remained edematous, and the graft was not detected on anterior segment optical coherence tomography. Subsequently, removal of the dislocated IOL, scleral fixation of a three-piece IOL (Avansee AN6KA; Kowa Company, Ltd., Japan), and Descemet stripping automated endothelial keratoplasty (DSAEK) were performed. Early posterior segment surgery was not initially pursued because there were no clinical or imaging findings suggestive of posterior segment involvement. The clinical priority was to restore corneal clarity and visual potential, and therefore, secondary DSAEK was performed. We attempted to identify the dropped DMEK graft intraoperatively during the secondary DSAEK using endoillumination under the vitrectomy system; however, the graft could not be visualized. The DMEK graft was again not identified intraoperatively.

**Figure 2:**
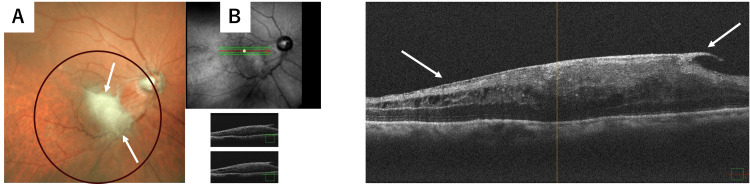
Macular proliferative changes detected after DSAEK. (A) Fundus photograph demonstrating localized proliferative vitreoretinal changes after DSAEK. (B) Optical coherence tomography showing hyperreflective proliferative tissue at the macula without evidence of retinal tears or hemorrhage. White arrows indicate the proliferative tissue at the macula. The black circle highlights an area without abnormal findings.

Following DSAEK, fundus examination revealed localized whitish proliferative vitreoretinal changes at the macula (Figure 2A). Optical coherence tomography further demonstrated a localized hyperreflective proliferative tissue over the macular region, without evidence of retinal breaks, intraretinal cystoid spaces, or hemorrhage (Figure 2B).

Suspecting posterior migration of the DMEK graft, pars plana vitrectomy was performed. Intraoperatively, a semi-transparent membranous tissue consistent with a migrated DMEK graft was identified in the inferior vitreous cavity, adherent to the macula and surrounded by fibrovascular tissue. The graft was removed en bloc without complications.

**Figure 3:**
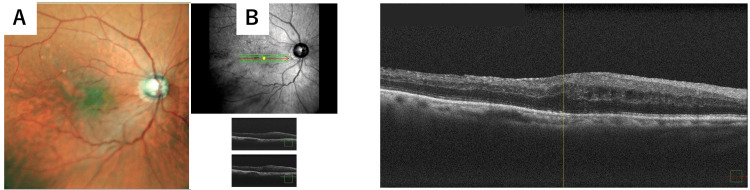
Resolution of macular proliferative changes after pars plana vitrectomy. (A) A fundus photograph after vitrectomy showing resolution of proliferative vitreous changes at the macula. (B) Optical coherence tomography demonstrating resolution of macula architecture following surgical intervention.

After pars plana vitrectomy and removal of the migrated graft, fundus examination demonstrated resolution of the macular proliferative changes (Figure 3A). Optical coherence tomography confirmed improvement of the macular architecture (Figure 3B). Post vitrectomy, best-corrected visual acuity was 20/100 in the right eye and 20/20 in the left eye. The intraocular pressure was 14 mmHg in the right eye and 17 mmHg in the left eye.

## Discussion

Posterior dislocation of a Descemet membrane endothelial keratoplasty (DMEK) graft is an exceedingly rare complication, with only a small number of cases reported in the literature to date [5-7]. Most published reports have focused primarily on the technical aspects of graft migration and retrieval, while the potential impact on the posterior segment has received limited attention. The present case broadens the clinical spectrum of this complication by demonstrating early localized macular proliferative changes associated with a migrated DMEK graft in a vitrectomized eye.

Several risk factors for posterior graft migration have been proposed, including prior vitrectomy, aphakia or intraocular lens (IOL) instability, large pupil diameter, and compromised anterior segment support [6,8]. In the current case, subtle IOL instability was present preoperatively but was difficult to evaluate because of poor pupillary dilation. This highlights a practical limitation in preoperative assessment and underscores the need for heightened caution when performing DMEK in eyes with complex surgical histories, particularly those with altered anterior and posterior segment anatomy. In addition, eyes with PEX syndrome, as in the present case, require careful consideration of surgical strategy because of potential zonular weakness and increased procedural risk.

Compared with Descemet stripping automated endothelial keratoplasty (DSAEK), DMEK grafts are thinner and lack stromal tissue, a characteristic generally believed to reduce fibrotic or inflammatory responses when dislocated into the vitreous cavity. Vasquez-Perez et al. performed histopathologic analysis of a posteriorly dislocated DMEK graft and demonstrated minimal cellular proliferation and limited fibrotic transformation, supporting the concept that DMEK tissue is biologically less reactive than DSAEK tissue [5]. In contrast, posterior dislocation of DSAEK grafts has been associated with retinal detachment and proliferative vitreoretinopathy (PVR), presumably due to the presence of stromal tissue and higher cellular content [7,9].

Despite these differences, the present case suggests that even a thin and largely acellular DMEK graft may contribute to clinically significant posterior segment changes under certain conditions. The macular findings in this patient were characterized by localized proliferative changes without evidence of retinal tears, hemorrhage, or ischemia. This presentation differs from classical PVR associated with rhegmatogenous retinal detachment and suggests a distinct pathogenic process rather than a conventional tractional or ischemic mechanism [9].

One possible explanation is that the migrated DMEK graft acted as a scaffold within the vitreous cavity, facilitating localized cellular proliferation or inflammatory activation near the macula. Experimental and clinical studies have shown that intraocular biomaterials and membranes can serve as substrates for cellular migration and proliferation within the vitreous cavity [9,10]. Alternatively, postoperative inflammatory responses or subclinical vitreoretinal traction may have contributed to the observed changes. These hypotheses remain speculative, and the exact biological mechanism underlying this phenomenon has yet to be elucidated.

Management of posteriorly migrated endothelial grafts remains controversial. Some authors advocate early surgical retrieval to prevent secondary complications [6], whereas others have reported favorable outcomes with observation when no posterior segment pathology is detected [5]. In the present case, progressive macular changes prompted pars plana vitrectomy, during which the migrated graft and associated proliferative tissue were successfully removed. Subsequent improvement in macular morphology suggests that timely intervention may be beneficial when posterior segment involvement becomes clinically evident.

This case also provides an important diagnostic lesson. When a DMEK graft cannot be visualized in the anterior chamber postoperatively, evaluation should not be limited to the anterior segment alone. In vitrectomized eyes, posterior migration may occur silently, and early posterior segment imaging - including optical coherence tomography and ultrasonography - should be considered to localize the graft and detect secondary complications [4,8].

Limitations

Several limitations should be acknowledged. This report describes a single case, which limits generalizability and precludes definitive causal inference between the migrated graft and macular changes. Histopathological analysis of the retrieved graft was not performed, preventing confirmation of cellular composition or inflammatory activity. Additionally, multiple confounding factors - including prior vitrectomy, IOL instability, uveitis, and advanced age - may have contributed to both graft behavior and retinal response. Ultrasonographic evaluation was not performed to localize the dropped DMEK graft prior to the secondary DSAEK surgery, which may have limited early detection and prevention of subsequent proliferative reactions. Finally, the precise mechanism underlying the macular proliferative changes remains unclear and warrants further investigation through the accumulation of similar cases and experimental studies.

## Conclusions

In conclusion, posterior migration of a DMEK graft, although rare, may be associated with clinically meaningful posterior segment complications. Awareness of this possibility is particularly important in eyes with prior vitrectomy or compromised anterior segment support. Early recognition, comprehensive posterior segment evaluation, and timely multidisciplinary management involving both corneal and retinal specialists may help prevent irreversible visual impairment and optimize outcomes following endothelial keratoplasty.
